# Effects of discontinuation of serotonergic antidepressants prior to psilocybin therapy versus escitalopram for major depression

**DOI:** 10.1177/02698811241237870

**Published:** 2024-03-22

**Authors:** David Erritzoe, Tommaso Barba, Meg J Spriggs, Fernando E Rosas, David J Nutt, Robin Carhart-Harris

**Affiliations:** 1 Division of Psychiatry, Department Brain Sciences, Centre for Psychedelic Research, Imperial College London, London, UK; 2 Department of Informatics, University of Sussex, Brighton, UK; 3 Departments of Neurology and Psychiatry, University of California San Francisco, San Francisco, CA, USA

**Keywords:** Depression, psychiatry, SSRI, escitalopram, psilocyibin

## Abstract

**Background:**

There is growing evidence for the therapeutic effects of the psychedelic drug psilocybin for major depression. However, due to the lack of safety data on combining psilocybin with selective serotonin reuptake inhibitors (SSRIs) and serotonin-norepinephrine reuptake inhibitors (SNRIs) and concerns that there may be a negative interaction on efficacy, participants enrolling in psychedelic trials are usually required to discontinue SNRI/SNRIs prior to enrolling.

**Aims:**

Using data from a recent clinical trial examining the comparative efficacy the psychedelic drug psilocybin (P) combined with approximately 20 h of psychological support to a 6-week (daily) course of the SSRI escitalopram plus matched psychological support for major depressive disorder, we explored the effects of discontinuing SSRI/SNRIs prior to study enrolment on study outcomes.

**Methods:**

Exploratory post hoc analyses using linear mixed effects model were performed to investigate the discontinuation effect on various validated depression symptom severity scales and well-being. The impact of SSRI/SNRIs discontinuation on the acute psychedelic experience was also explored.

**Results/outcomes:**

In the psilocybin group, there was a reduced treatment effect on all outcome measures for SSRI/SNRIs discontinuers compared with unmedicated patients at trial entry. However, no effects of discontinuation on measures of the acute psychedelic experience were found.

**Conclusion:**

Discontinuation of SSRI/SNRIs before psilocybin might diminish response to treatment; however, as we did not test SSRI/SNRI continuation in our trial, we cannot infer such causation. Moreover, the exploratory nature of the analyses makes them hypothesis generating, and not confirmatory. A controlled trial of SSRI/SNRI discontinuation versus continuation prior to psilocybin is urgently required.

## Introduction

Major depressive disorder (MDD) is one of the most economically and socially burdensome diseases worldwide (Friedrich, 2017) and involves marked changes in mood, motivation, pleasure and cognition (Otte et al., 2016). MDD is the second leading contributor to chronic disease burden among all medical conditions, as measured by ‘years lived with disability’ (James et al., 2018) and each year affects about 6% of the worldwide adult population (Bromet et al., 2011). First-line treatment for MDD typically involves pharmacotherapy, which may or may not be coupled with psychotherapy. The most widely used medications for MDD are selective serotonin reuptake inhibitors (SSRIs) and serotonin-norepinephrine reuptake inhibitors (SNRIs). While serotonin reuptake inhibitors (SRIs) generally have greater tolerability compared to their older counterparts, side effects are common (Cipriani et al., 2009). Moreover, they do not induce a response in all patients (Cipriani et al., 2018). There is thus a great need for new efficacious treatment options.

The classic psychedelic drug psilocybin has recently been investigated as a possible alternative treatment option for MDD. Psilocybin, through agonism of serotonin 2A receptors (5-HT_2A_R), is believed to target rigid pathological belief systems by increasing psychological and neurobiological flexibility (Carhart-Harris et al., 2023; Carhart-Harris and Friston, 2019; Carhart-Harris and Nutt, 2017) This ‘flexible’ brain state is believed to facilitate emotional release and psychological insights that often arise during and after the acute psychedelic experience. Furthermore, when given in a therapeutic setting, these acute subjective effects have been shown to be facilitative of greater long-term positive outcomes (Carhart-Harris and Friston, 2019; Murphy et al., 2021; Peill et al., 2022).

A growing number of clinical trials have found prolonged reductions in the clinical symptoms of MDD following the administration of one-to-three doses of psilocybin in a therapeutic setting (Carhart-Harris et al., 2021, 2016; Davis et al., 2021; Gukasyan et al., 2022). Carhart-Harris et al. (2021) recently presented the first double-blind randomized control trial to compare psilocybin with a leading SSRI medication, escitalopram, in individuals diagnosed with MDD. Based on the study’s primary outcome measure, the Quick Inventory of Depression Symptomology Self-Report Version (QIDS-SR16), two doses of psilocybin were found to elicit nominally greater reductions in depression symptoms than the 6-week course of escitalopram, but not to a statistically significant extent. However, psilocybin outperformed escitalopram on all secondary outcome measures including all of the other depression rating scales as well as measures of well-being, anhedonia, social functioning, rumination, sexual functioning and emotional responsivity (Barba et al., 2022; Carhart-Harris et al., 2021).

As is standard practice in psychedelic trials, participants in the Carhart-Harris et al.’s (2021) study, who were taking SSRI/SNRIs at screening, were required to discontinue prior to commencing the study. This practice is due to some overlap in serotonergic pharmacology of SSRI/SNRIs and psilocybin (i.e. both cause elevated stimulation of 5-HT receptors) as well as an absence of established safety data for combined treatment – at least at the time of conducting the present study (Malcolm and Thomas, 2021). Additionally, previous case reports have indicated that long-term administration of SSRI/SNRIs can reduce the therapeutically important subjective effects of psychedelics (Bonson et al., 1996; Strassman, 1992). In accordance with guidelines set forth by the National Institute for Health and Care Excellence (NICE; National Institute for Health and Care Excellence, 2022) and the British Association for Psychopharmacology (Cleare et al., 2015), SSRI/SNRI tapering in this study was done gradually for a period of 2–4 weeks with linear reductions to minimize withdrawal symptoms. There was at least a two-week (or 5 half-lives) drug-free washout period before study commencement, and participants were required to remain abstinent from the medication for the duration of the trial (Carhart-Harris et al., 2021; Carhart-Harris et al., 2016; Davis et al., 2021; Ross et al., 2016). Once the trial was complete, participants could re-start antidepressant medication under the guidance of their main care provider, typically their general practitioner (GP). This process has been found to be safe and effective (i.e. no serious adverse events) in clinical trials thus far (Carhart-Harris et al., 2016; Carhart-Harris et al., 2021).

For some individuals, however, stopping treatment with SSRI/SNRIs can cause various unpleasant physiological and psychological symptoms such as nausea, sleep problems, lethargy, dysphoric mood, paraesthesia and dizziness. Although some of these symptoms overlap with symptoms of MDD, this “discontinuation syndrome” is distinct from a relapse (American Psychiatric Association, 2013) and may last up to 6 weeks, or even up to 12 weeks for some patients (Horowitz and Taylor, 2019; Lerner and Klein, 2019). Symptom usually resolve by reintroducing SSRI/SNRIs (Bhat and Kennedy, 2017; Fava et al., 2015; Warner et al., 2006). Previous research pooling data from small scale studies of the related psychedelic compound 3,4-methylenedioxy methamphetamine (MDMA) showed that discontinuing reuptake inhibitor antidepressant medications (versus no immediate prior use of such medications) had a negative impact on study outcomes in the treatment of post-traumatic stress disorder (PTSD) – meaning that those who were unmedicated before the start of the trial responded better to MDMA (Feduccia et al., 2021). The effects of discontinuing SSRI/SNRIs on study outcomes have yet to be formally tested in trials of psilocybin and other classic psychedelics. Moreover, it is important to note that the interplay between patient expectations and clinical outcomes is a complex and critical element in the design and interpretation of medical trials. This becomes even more pronounced in studies involving psychotropic substances, such as psychedelics, where subjective experiences and personal beliefs can markedly influence patient responses (Muthukumaraswamy et al., 2021). In light of this, it becomes essential to explore how preconceived notions about treatment, particularly among those with prior experiences with SSRI/SNRIs, might shape the outcomes of such trials.

The aim of the current paper was this to explore a potential “discontinuation effect” of SSRI/SNRIs titration prior to study commencement in the Carhart-Harris et al., (2021) trial. Specifically, we investigated through exploratory *post hoc* analyses (inspired by published comments to Carhart-Harris et al., (2021)) for possible differences in outcomes in participants who did and did not discontinue SSRI/SNRIs (venlafaxine, paroxetine, sertraline, duloxetine and citalopram) at the start of the trial (referred to as “discontinuers” and “unmedicated” respectively). Additionally, we explored if baseline expectations of response to treatment affected outcomes in either group.

## Methods

### Study outline

#### Design

For full explanation of the study design, see Carhart-Harris et al., (2021). Briefly, this double-blind randomized control trial (RCT) compared psilocybin (as COMP360) to the SSRI escitalopram in participants with a diagnosis of moderate-severe major depression (MDD). Fifty-nine participants received two oral doses of psilocybin accompanied by psychological support, separated by 3 weeks. Those in the “psilocybin” condition (N = 30; females = 11) received two 25 mg doses of psilocybin and a 6-week course of placebo. Those in the “escitalopram” condition (N = 29; females = 9) received two 1 mg (placebo-like) doses of psilocybin coupled with a 6-week course of escitalopram. Taking into account screening, preparation, dosing and integration, participants in each condition received approximately 20 hours of in-person therapeutic support during the trial, as well as up to six further integration calls over Skype or by telephone. There was no difference between conditions in the adoption of these optional calls (Carhart-Harris et al., 2021).

#### Participants

All participants provided written informed consent after being provided with a complete description of the study. Inclusion criteria included an age range of 18–85 years, physically healthy (determined via physical examination) and a diagnosis of unipolar MDD (as confirmed by a GP). Key exclusion criteria included diagnosis of a psychotic disorder, immediate family member with a psychotic disorder, positive pregnancy test at screening or during the study, excessive alcohol or drug use and MRI contraindications. For a full list of eligibility criteria, see Carhart-Harris et al., (2021).

Participants who were taking serotonergic medication at screening but were otherwise deemed eligible were required to discontinue their serotonergic medication before baseline measures were collected. This took place in the weeks prior to the first study visit. Discontinuation procedures were managed through collaboration between the participants prescribing GP and the psychiatrists on the study team.

#### Intervention

For full details on the therapeutic approach, refer to Carhart-Harris et al. (2021), Watts et al. (2020) and Murphy et al. (2022). Briefly, each participant was paired with two ‘guides’ (therapists, or psychiatrists with therapy experience) who they worked with for the duration of the trial. During dosing, participants were encouraged to recline in a semi-supine position and were provided with an eye mask and headphones. Dosing days were preceded by psychological preparation (building the therapeutic relationship and discussing what to expect from the experience) and integration (non-judgemental listening to the participants’ experience, aiding their ability to contextualize and assimilate it). This approach is consistent with standard psychedelic-therapy protocols (Johnson et al., 2008).

#### Regulatory oversight

The trial received favourable opinion from the Brent National Research Ethics Service and was reviewed and approved by the Health Research Authority and Medicines and Healthcare products Regulatory Agency. The trial was sponsored by Imperial College London’s Joint Research and Compliance Office and was adopted by the National Institute of Health Research (NIHR) Clinical Research Network. The study took place at the NIHR-funded Imperial College Research Facility. Psilocybin was provided by COMPASS pathways, and escitalopram and placebo were provided by the Pharmacy Manufacturing Unit at Guy’s and St Thomas’s Hospital.

### Aims

The overall goal of this investigation is to evaluate the overall effect of recent SSRI/SNRI discontinuation on depression outcomes. For this, we explored the following four sub-aims. (1) We examined differences in baseline symptomology as well as changes in depression severity from screening (i.e. before discontinuation started) to baseline (i.e. after discontinuation) in discontinuers and unmedicated patients. (2) We assessed whether response to treatment based on the Quick Inventory of Depressive Symptomology (QIDS-SR16; primary outcome measure of the trial) differed *for SSRI/SNRIs discontinuers and unmedicated patients separately*. This analysis expands on the primary analysis presented by (Carhart-Harris et al., 2021), and answers the question ‘does response to treatment differ between discontinuers and unmedicated patients at trial start?’ To further determine the consistency of potential discontinuation effects within treatment conditions, we also assessed the following secondary depression and well-being measures^1^ in the same manner: Beck Depression Inventory (BDI), Hamilton Rating Scale for Depression (HAM-D), Montgomery Äsberg Depression Rating Scale (MADRS) and psychological well-being (WEMWBS). (3) For gaining additional insight, we then assessed whether treatment response differed between SSRI/SNRIs discontinuers and unmedicated *between the two treatment conditions* to determine whether discontinuation differentially shapes response to either psilocybin or escitalopram. This further analysis was performed in order to answers the question ‘do discontinuers and unmedicated patients respond differently within each condition?’ For this analysis, we focused on the study’s primary outcome, the QIDS-SR16. (4) We further explored whether discontinuation attenuated known psychological modulators of therapeutic outcomes of psychedelic-assisted therapy, including widely used acute indices in the domains of mystical-type experience, challenging experience emotional breakthrough and ego dissolution. Finally, we also explored the impact of expectation, which has been argued to be capable of playing an important role in psychedelic trials (Kaertner et al., 2021; Muthukumaraswamy et al., 2021).

### Outcome measures

#### Psychological outcomes

For the purposes of this study, four measures of depression were analysed. Two of these measures are widely used self-report measures of depression: (1) QIDS-SR16 (Rush et al., 2003), which acted as the primary outcome measure of Carhart-Harris et al. (2021) and was administered weekly for the course of the trial and (2) the Beck Depression Inventory 1A (BDI-1A; Beck et al., 1996), which was administered bi-weekly for the course of the trial. The remaining two scales are clinician administered measures of depression symptomology that were administered at baseline and follow-up: (1) the Hamilton Depression Rating Scale (HAM-D) (Hamilton, 1960) and (2) the Montgomery and Asberg Depression Rating Scale (MADRS) (Montgomery and Asberg, 1979). In addition to these clinical measures of depression, we also include an analysis of the Warwick-Edinburgh Mental Well-being Scales (WEMWBS; Tennant et al., 2007) which was a key secondary outcome measure of Carhart-Harris et al. (2021) and was measured bi-weekly for the course of the trial.

#### Acute experience

As potential modulators of outcome, we also analysed four measures of the acute experience: the *mystical experience questionnaire* (MEQ; Barrett et al., 2015), the *ego dissolution inventory* (EDI; Nour et al., 2016), the *emotional breakthrough inventory* (EBI; Roseman et al., 2019), and the *challenging experience questionnaire* (CEQ; Barrett et al., 2016). These measures were chosen due to their wide use in psychedelic research, and because they index different aspects of the acute experience that are thought to play an important role in the therapeutic efficacy of psilocybin.

#### Expectation

Expectation was assessed with two questions at baseline. Specifically, participants were asked to rate (on a scale from 0 to 100) how much they expected their mental health to improve after receiving (1) 6 weeks of escitalopram, (2) 2 doses of psilocybin 3 weeks apart. All participants were asked both questions, irrespective of the arm they were randomized to.

### Statistical analysis

All analyses were conceived of post hoc and in response to comments to Carhart-Harris et al. (2021). Analyses were performed in R Studio (www.rstudio.com/) using the packages lme4 (Bates et al., 2015), lmertest and ggplot2. The results of significance testing (i.e. *p* values) are reported where these are required to meet the aim of the test performed, that is, for demographics (where the aim is to test for a difference between groups) and for model comparisons (where the aim is to determine the winning model). We reported effect sizes, and 95% confidence intervals (95% CI) when the aim was to assess the magnitude of the discontinuation effect, (Cumming et al., 2012). Full results (including *p* values) can be found in Supplemental Materials. Given the exploratory post hoc nature of the analyses and the high correlations between the different depression measures used in this study (Li et al., 2017), we did not correct the results for multiple comparisons.

#### Differences in demographics

T-test and Chi-square tests were performed on demographic variables to assess for differences between the psilocybin and escitalopram conditions. Additionally, a Chi-square test was used to assess differences in the proportion of discontinuers and unmedicated patients in each treatment condition.

#### Effect of discontinuation on response to treatment

To assess whether response to treatment differed for discontinuers and unmedicated patients separately (Aim 1), mixed effects linear models were fit separately for those who discontinued, and those who did not for each depression measure. In both the cases, two models were specified that took the forms:

M_1_: *Outcome–Timepoint* + (1|*Participant*)
M_2_: *Outcome–Timepoint* * *Treatment* + (1|*Participant*)

where ‘*Outcome*’ is the psychological scale, ‘*Treatment*’ is the treatment condition (Psilocybin or Escitalopram) and ‘*Timepoint*’ is the timepoint the measure was taken, which differs for the five outcome measures (*weekly* for QIDS-SR16, *bi-weekly* for BDI and WEMWBS, and *Baseline and Final Follow-up (6 weeks)* for HAMD and MADRS). Both Treatment and Timepoint are treated as fixed effects, and participant was considered as a random intercept.

To determine whether treatment condition adds significantly to the model (i.e. whether there was a consistent difference between the psilocybin arm and the escitalopram arm), an analysis of variance was performed between M_1_ and M_2_, where M_1_ is thought of as the null mode, and M_2_ as the alternative. As it is known that people improve over time in both treatment arms (shown in Carhart-Harris et al., 2021), timepoint is still part of the null model. A significant difference indicates that the treatment condition adds consistently to the model.

In cases where the outcome of model comparison is significant, we then explore M_2_ to assess the direction and magnitude of the effect of the term *Treatment*. In cases where there is no significant difference, no further exploration is performed. Full results of M_2_ can be found in Supplemental Materials.

In all models, *escitalopram* is treated as the reference condition, that is, results for the effect of timepoint alone are related to the subjects in the escitalopram arm. As we are interested in the difference between the two treatment arms, we will focus our interpretations on the interaction terms involving *Treatment*.

#### Response to treatment based on discontinuation

To additionally explore whether responses in each treatment arm were different for those who had recently discontinued from serotonergic medication (Aim 2), a linear mixed effects model was defined with the QIDS-SR16 as the outcome. This model took the form

M_3_: *Outcome–Timepoint* * *Treatment* * *Discontinuation* + (1|*Participant*),

where ‘*Timepoint*’ is a categorical variable considering each timepoint of the study, ‘*Treatment*’ is a categorical variable considering escitalopram or psilocybin arms, ‘*Discontinuation*’ is a categorical variable that refers to whether participants discontinued or did not discontinue prior to participation and ‘*Participant*’ is a categorical variable that refers to the identity of each subject. In this model, the reference conditions are *baseline* (for Timepoint), *escitalopram* (for Treatment) and *unmedicated* (for Discontinuation). Treatment, Timepoint and Discontinuation are treated as fixed effects, and participant is considered as a random intercept.

Here, we focus our interpretation on two interactions. Firstly, the *Time:Discontinuation* interaction reveals whether discontinuation modulates response from baseline to another timepoint within the reference condition escitalopram. Specifically, the parameter estimate is the mean value of the difference in the observed QIDS-SR16 change from baseline for those who did discontinue in the escitalopram arm with respect to those who did not. Secondly, the *Time:Discontinuation:Treatment* interaction reveals whether discontinuation modulates QIDS-SR16 change within the psilocybin arm. That is, the parameter estimates here represent the difference in QIDS-SR16 change from baseline for those who did discontinue in the psilocybin arm. Together, these two interactions, therefore, reveal how discontinuation differentially shaped response between the two treatment arms.

#### Calculation of effect sizes

To assess the magnitude of various changes of interest in the various mixed models, we employed two complementary approaches. First, all models are implemented using raw scores, and hence, the estimates, their standard error and their confidence intervals can be readily interpreted directly (i.e. an estimate of *x* units imply that under that condition subjects on average display *x* more units than the corresponding baseline). Secondly, we estimate effect sizes for the estimates obtained via linear mixed models, with provide unit-less quantities that characterize the ratio between differences between subpopulation means and the standard deviation of the error. For this purpose, we follow the approach outlined in (Brysbaert and Stevens, 2018; Westfall et al., 2014) and calculate *parameter effect sizes*, which are given by the ratio between the estimate and the pooled variance, which in our case corresponds to

$$d=\frac{\beta }{\sqrt{\sigma_{p}^{2}+\sigma_{e}^{2}}},$$

where $\sigma_{p}^{2}$ is the variance of the random intercept assigned to participants, and $\sigma_{e}^{2}$ is the variance of the residuals of the model. That being said, it should be noted that – as discussed in the mentioned references – effect sizes in mixed models are less straightforward to interpret than in simpler designs. For simplicity, given the nuanced dependency between parameter effect sizes of simple effects computed in this manner and their coding (as highlighted in (Westfall et al., 2014)), we only report parameter effect sizes of interactions in models M_2_.

#### Effect of discontinuation on baseline depression scores

The impact of discontinuation on depression at baseline (Aim 4) was assessed using a simple regression model with depression scores at baseline as outcome (QIDS-SR16, BDI, HAM-D MDRAS) and discontinuation as predictor. Leveraging the fact that both QIDS-SR16 and BDI were collected at screening (i.e. before discontinuation started), a linear mixed model with discontinuation as a predictor assess change in depression scores from screening to baseline.

#### Modulators of response

##### Acute experience

To assess whether the acute experience modulated response (Aim 3), linear models were constructed using treatment and discontinuation for predicting the subjective scores in four key indicators of the psychedelic experience: the MEQ (Barrett et al., 2015), the EDI (Nour et al., 2016), the EBI (Roseman et al., 2019) and the CEQ (Barrett et al., 2016). Models with and without interaction were constructed, and model selection was then performed using the Akaike information criteria.

##### Expectation

To evaluate if baseline differences in expectation impact any discontinuation effect (Aim 4), we ran a linear regression using expectation to the efficacy of psilocybin or escitalopram to treat depression as target, and discontinuation as predictor.

## Results

### Differences in demographics

T-test and Chi-square tests were performed on the psilocybin condition and escitalopram condition separately. There were no significant difference in age (*t*_(57)_ = −0.403, *p* = 0.35), sex (*χ*^2^_(1, *N*__=__59)_ = 2.6, *p* = 0.1), ethnicity (*χ*^2^_(8, *N*__=__59)_ = 7.04, *p* = 0.53) or employment status (*χ*^2^_(4, *N*__=__59)_ = 2.8, *p* = 0.64) between discontinuers and unmedicated patients.

Of those who discontinued medication prior to entering the trial, 11 patients in the psilocybin group and 9 in the escitalopram group were on SSRI/SNRIs (venlafaxine, paroxetine, sertraline, duloxetine and citalopram). One patient in the psilocybin group and 3 in the escitalopram group were on other psychiatric medications (mirtazapine, pregabalin, bupropion and bupropion extended release). Patients who discontinued these other psychiatric medications were not included in further analyses. All patients completely discontinued medications at least 2 weeks before the start of the trial. There was no difference in the proportion of participants who discontinued in each arm (*χ*^2^_(1, *N*__=__59)_ = 4.932, *p* = 0.1) (Table 1).

**Table 1.** Demographics. For detailed demographics, refer to Carhart-Harris et al. (2021).

Treatment	Discontinuers (*N* = 21)		Unmedicated (*N* = 35)	
	Psilocybin (*N* = 11)	Escitalopram (*N* = 9)	Psilocybin (*N* = 18)	Escitalopram (*N* = 17)
N				
Total	11	9	19	19
Females	2	3	9	6
Age				
*M* (SD)	46.3 (11.2)	35.3 (7.5)	41.6 (12.0)	41.1 (10.4)
Range	21–64	22–46	24–61	24–60
Ethnicity				
White *N*	10	10	18	14
No. of psychiatric medications previously used				
*M* (SD)	3.1 (.4)	2.3 (0.4)	1.6 (.3)	1.5 (0.4)
Range	1–6	1–4	0–5	0–5

### Effect of discontinuation on depression scores at baseline

**Figure 1.**
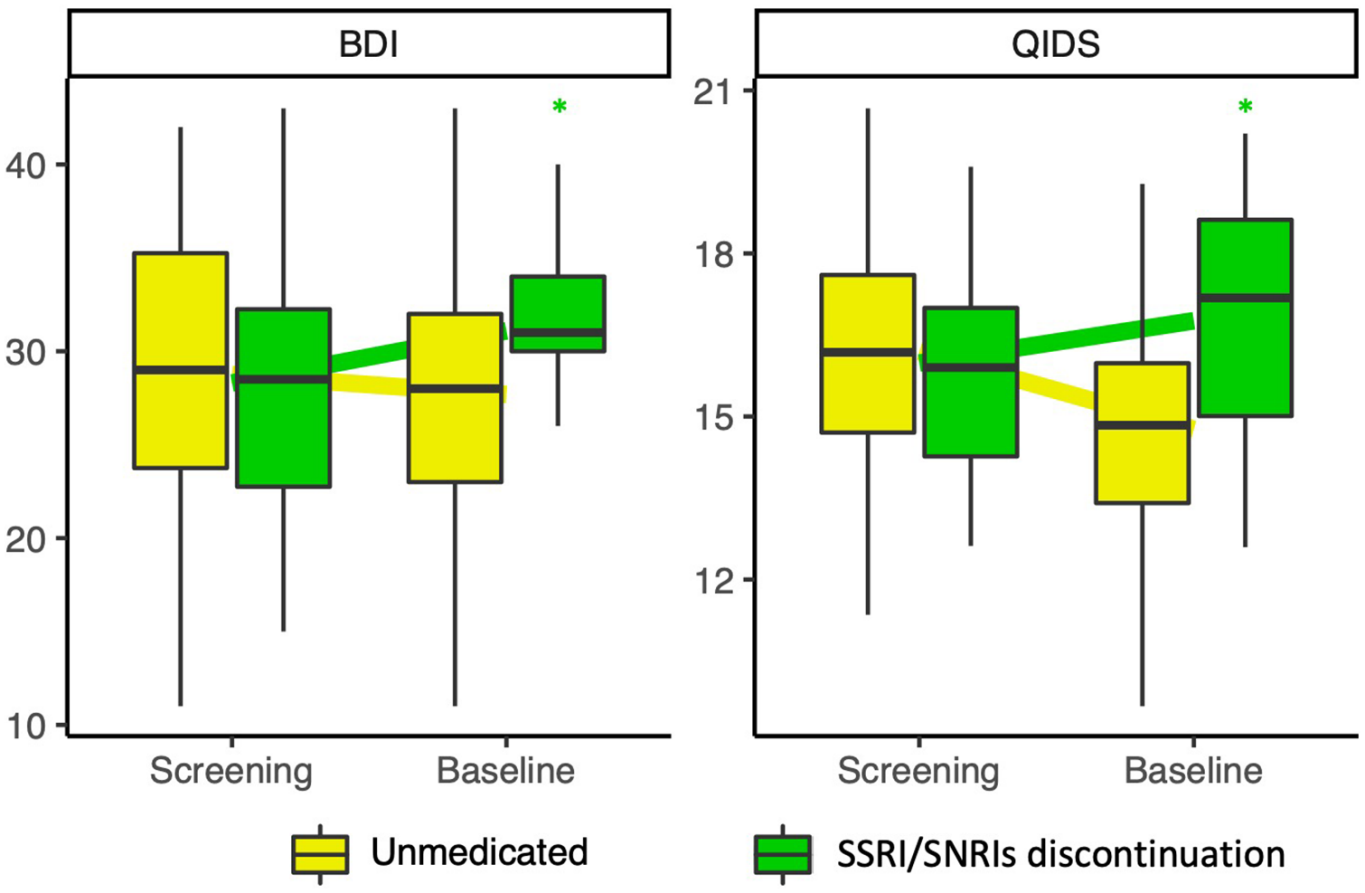
BDI (right) and QIDS-SR-16 (left) scores at screening and baseline for those who did and did not discontinue SADs separately. QIDS-SR16 and BDI scores increased from screening to baseline in those who discontinued (Yellow) compared to those who were unmedicated coming into the trial (Green). *Indicates univariate significance *p* < 0.05.

Regression modelling on baseline depression scores (i.e. just before starting study treatment) with discontinuation as predictor revealed a trend towards greater depression scores at baseline in those who had discontinued compared to those who had not (QIDS-SR16 β = 2.00, 95% CI (−0.18, 4.18); HAMD β = 1.99, 95% CI (0.50, 3.48); MADRS β = 2.25, 95% CI (−0.24, 4.74); BDI β = 3.14, 95% CI (−0.79, 7.06)). Linear mixed modelling revealed a significant increase from screening to baseline in QIDS-SR16 scores (Screening mean 15.95 (3.44), baseline mean 16.75 (4.05)) and BDI scores (Screening mean 28.30 (7.34), baseline mean 31.10 (4.70)) in those who discontinued compared to those who were unmedicated (QIDS-SR16 β = 2.21, 95% CI (0.21, 4.23), BDI β = 3.66, 95% CI (0.85, 6.48)), suggesting a negative effect on depression severity of discontinuation before the start of the trial (Figure 1, Supplemental Table S1). The QIDS data also suggest a nominal improvement in symptom severity from screening to baseline in the unmedicated.

#### Effect of discontinuation on response to treatment

For the following analyses, models were fit for unmedicated patients and discontinuers separately. Full results can be found in Tables S3 (unmedicated patients) and S4 (discontinuers).

### QIDS-SR16

**Figure 2.**
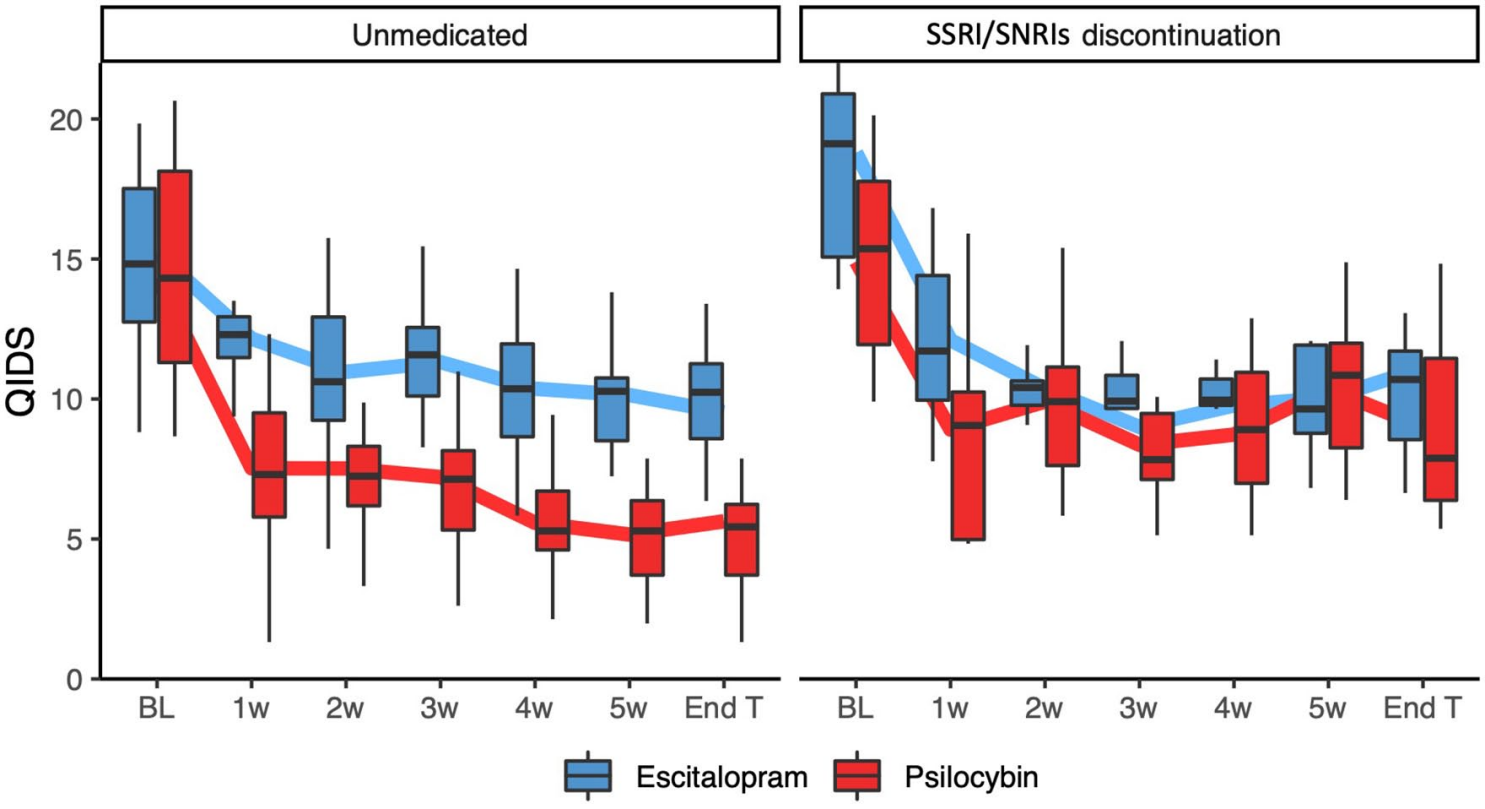
QIDS-SR16 scores across time for discontinuers and unmedicated patients separately, after removing the effect of individual differences as captured by a random intercept in a mixed linear model. Thick lines connect mean values, and boxes show medians, 25 and 75 percentiles. Among unmedicated patients, greater decreases from baseline were seen in the psilocybin group compared with the escitalopram group at all timepoints except week 2. Among discontinuers, being in different treatment arms did not significantly add to the model.

Model comparison for discontinuers revealed no significant difference between M_1_ and M_2_ $\left(\chi^{2}(8)=\;7.45,\;p\;=\;0.489\right)$ , indicating that *Treatment* did not add significantly to the model (Table S4). Conversely, there was a significant difference between M_1_ and M_2_ for the unmedicated $\left(\chi^{2}(8)=\;21.39,\;p\;=\;0.006\right)$ (Table S3). As demonstrated in Figure 2, among unmedicated patients there were significant interactions between timepoint and condition with the psilocybin group, demonstrating greater decreases from baseline in QIDS-SR16 compared with the escitalopram group at all timepoints except week 2 (Week 1 β = −3.73, 95% CI (−6.40, −1.06); Week 3 β = −3.21, 95% CI (−5.81, −0.61); Week 4 β = −3.89, 95% CI (−6.56, −1.22); Week 5 β = −4.08, 95% CI (−6.73, −1.44); Week 6 β = −3.39, 95% CI (−6.01, −0.76); Follow-up β = −3.00, 95% CI (−5.60, −0.40)). Across the significant timepoints, the magnitude of decrease in QIDS-SR16 for the psilocybin group (on top of the decrease seen for escitalopram) was an additional three to four QIDS-SR16 points, which corresponds to an additional antidepressant effect of 50%–120%.

#### Additional depression and well-being measures

**Figure 3.**
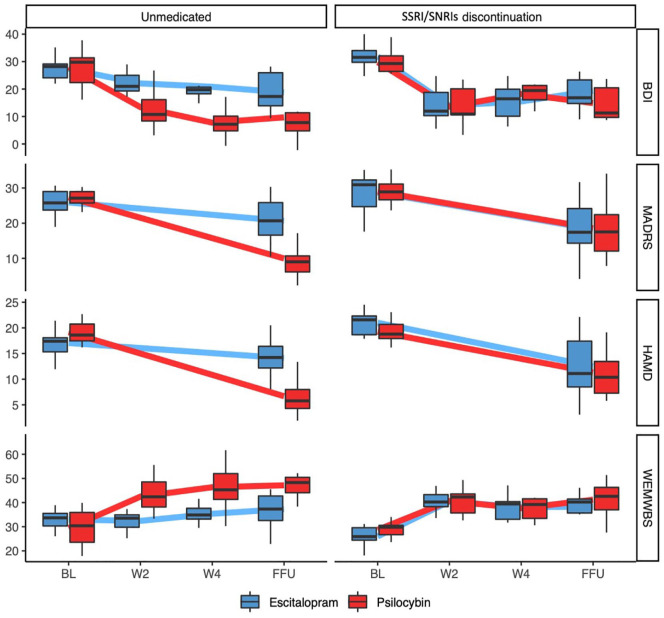
Scores across time for unmedicated on entry and SAD discontinuers for the following scales (from top to bottom): HAM-D, MDRAS, BDI and WEMWBS. Thick lines connect mean values, and boxes show medians, 25 and 75 percentiles. For the unmedicated on entry, greater response was seen in the psilocybin group compared with escitalopram. For discontinuers, being in different treatment arms did not significantly add to the model. BL: baseline; W2: week 2; W4: week 4; FFU: final follow-up.

Similarly to what was seen for QIDS-SR-16, for all the additional depression measures, model comparison for discontinuers revealed no significant difference between M_1_ and M_2_ (HAM-D: $\chi^{2}\left(2\right)=\;1.06\;,\;p\;=\;0.589$ ; MADRS: $\chi^{2}\left(2\right)=\;0.01,\;p\;=\;0.995$ ; BDI: $\chi^{2}\left(4\right)=\;2.83,\;p\;=\;0.586$ ), indicating that being in different treatment arms did not add significantly to the model. In contrast, significant differences between M_1_ and M_2_ were found for all measures for the unmedicated patients on entry *(*HAM-D: $\chi^{2}\left(2\right)=\;36.60\;,\;p<.001$ ; MADRS: $\chi^{2}\left(2\right)=26.66\;,\;p<\;.001$ ; BDI: $\chi^{2}\left(4\right)=\;27.14\;,\;p<\;.001$ ). For all scales, M_2_ is summarized in Figure 3 (Tables S2 and S3).

While investigating changes in unmedicated patients, for the HAM-D there was a significant negative interaction between *Timepoint* and *Treatment*, with those in the psilocybin arm showing an additional drop of 9.52 points (323%) compared with the drop in escitalopram group (95% CI (−12.43, −6.62)). The MADRS showed a similar negative interaction between *Timepoint* and *Treatment*, with those in the psilocybin arm showing an additional drop of 12.08 (229%) points compared with the escitalopram group (95% CI (−16.87, −7.30)). Finally, the BDI also showed a negative interaction between *Timepoint* and *Treatment*, with those in the psilocybin condition showing consistently greater drops in scores from baseline as compared to escitalopram (Week 2 β = −9.25, 95% CI (−15.52, −2.99); Week 4 β = −13.68, 95% CI (−19.94, −7.41); Follow-up β = −9.68, 95% CI (−15.94, −3.41)). As seen from BDI scores, the added drop in the psilocybin group over and above that seen in escitalopram corresponded to an additional antidepressant effect of 113%–210%.

When considering well-being scores (WEMWBS), model comparison for discontinuers revealed no significant difference between M_1_ and M_2_ $\left(\chi^{2}\left(4\right)=\;1.1,\;p\;=\;0.894\right)$ . As with depression scores too, there was a significant difference between M_1_ and M_2_ for the unmedicated on entry $\left(\chi^{2}\left(4\right)=\;33.46\;,\;p<\;.001\right)$ . M_2_ for the unmedicated on entry is summarized in Figure 3 (Tables S3 and S4). There was a significant negative interaction between *Timepoint* and *Treatment*, with those in the psilocybin condition showing consistently greater increases in WEMWBS from baseline as compared to escitalopram (Week 2 β = 13.58, 95% CI (7.40, 19.76); Week 4 β = 14.25, 95% CI (7.95, 20.54); Follow-up β = 13.42, 95% CI (7.24, 19.60)). The added increase in the psilocybin group over and above that seen in escitalopram ranged from 13.4 to 14.2 WEMWBS points – which cannot be mathematically estimated as a ratio in this case, given than at Week 2, there are zero positive changes in WEMWBS in the escitalopram arm.

#### Response to treatment based on discontinuation

Model M_3_ (i.e. the full model considering all subjects) was used to assess whether discontinuers and unmedicated responded differently when looking at the two treatment conditions separately (Table S4). A significant negative interaction between *Time* and *Discontinuation* was found at all weekly timepoints between weeks 1 and 6. This demonstrates that, in the escitalopram arm, greater decreases in QIDS-SR16 were seen for those who had discontinued, compared to the unmedicated (Week 1 β = −3.64, 95% CI (−7.04, −0.24); Week 2 β = −4.20, 95% CI (−7.60, −0.79); Week 3 β = −5.91, 95% CI (−9.18, −2.63); Week 4 β = −4.19, 95% CI (−7.59, −0.79); Week 5 β = −3.70, 95% CI (−7.07, −0.32)). A significant positive interaction between *Time, Treatment*, and *Discontinuation* was also found at all timepoints except week 1. This demonstrates that in the psilocybin condition, the anti-depressant response was reduced for those who did discontinue compared to those who were unmedicated coming into the trial (Week 2 β = 5.99, 95% CI (1.33, 10.66); Week 3 β = 6.47, 95% CI (1.91, 11.02); Week 4 β = 6.74, 95% CI (2.09, 11.39); Week 5 β = 8.31, 95% CI (3.67, 12.94); Week 6 β = 6.31, 95% CI (1.74, 10.89); Follow-up β = 4.79, 95% CI (0.23, 9.35)).

#### Effect of discontinuation on the acute experience

Regression models on the acute experience revealed that discontinuation was not a significant predictor for any of the acute indices measured (MEQ dosing 1: β = 0.04, 95% CI (−0.11, 0.18); MEQ dosing 2: β = −0.05, 95% CI (−0.20, 0.10); EDI dosing 1: β = 4.29, 95% CI (−11.14, 19.70); EDI dosing 2: β =−1.01, 95% CI (−17.78, 15.76); EBI dosing 1: β = 10.22, 95% CI (−5.43, 25.87); EBI dosing 2: β = 0.23, 95% CI (−17.55, 17.09); CEQ dosing 1: β = 0.002, 95% CI (−0.089, 0.093); CEQ dosing 2: β = 0.015, 95% CI (−0.083, 0.114]). In contrast, condition was a significant predictor in all models (MEQ dosing 1: β = 0.47, 95% CI (0.33, 0.60); MEQ dosing 2: β = 0.39, 95% CI (0.24, 0.53); EDI dosing 1: β = 34.50, 95% CI (0.24, 0.53); EDI dosing 2: β = 31.13, 95% CI (15.47, 46.79); EBI dosing 1: β = 39.49, 95% CI (24.67, 54.30); EBI dosing 2: β = 42.70, 95% CI (26.38, 59.04); CEQ dosing 1: β = 0.153, 95% CI (0.067, 0.239); CEQ dosing 2: β = 0.249, 95% CI (0.157, 0.341)). In effect, while the treatment always played a significant role in predicting acute measures, discontinuation did not. Furthermore, model comparison revealed no significant interactions between discontinuation and treatment condition (full model summaries are provided for the selected models in Supplemental Materials).

#### Expectation does not explain differences on psilocybin arm

**Figure 4.**
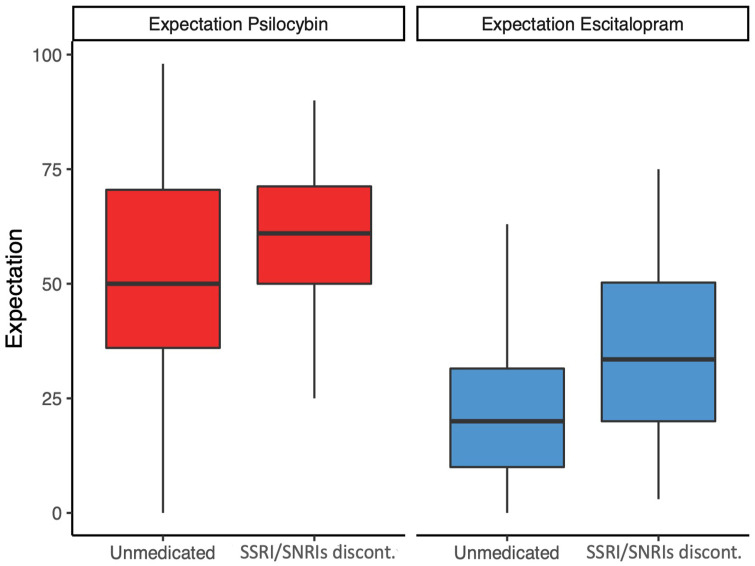
Baseline expectations for psilocybin and escitalopram for discontinuers and unmedicated patients separately. Boxes show medians, 25 and 75 percentiles. There is a trend for patients who discontinued having higher expectations for escitalopram.

Regressions assessing expectation at baseline revealed that while there was no statistically significant relationship between discontinuation and expectancy to psilocybin (β = 5.14, 95% CI (−7.27, 17.54)), there was a trend for patients who discontinued SSRI/SNRIs to have higher expectations for escitalopram pre-trial (β = 10.9, 95% CI (−0.09, 21.88)), as can be seen in Figure 4.

## Discussion

Here, we examined how pre-trial discontinuation of selective serotonin-reuptake inhibitors or serotonin noradrenaline reuptake-inhibitors affected outcomes in a 6-week double-bind randomized controlled trial comparing psilocybin with escitalopram in patients with longstanding, moderate-to-severe MDD. Our results build upon those in the response to a letter-to-the-editor by Carhart-Harris et al. (2021) and show that SSRI/SNRI medication use may have had a substantial effect on response to treatment, even when standard withdrawal procedures prior to study commencement are followed. In the present analyses, we found that unmedicated patients had a better response to psilocybin across multiple measures, unlike those who discontinued SSRI/SNRIs, who showed no significant difference. A larger analysis revealed that discontinuing these medications affected responses differently: discontinuers had a lesser response to psilocybin but responded better to escitalopram (Supplemental Table 4), suggesting that pre-treatment discontinuation of SSRI/SNRIs has contrasting impacts on treatment efficacy based on the type of medication administered.

These results provide a more nuanced interpretation of the results presented in (Carhart-Harris et al., 2021), which reported an absence of evidence for an effect of treatment condition in the primary outcome QIDS-SR-16 (despite there being between-subjects differences in other measures of depression, possibly linked to the presence of compound imprecise items in the primary outcome (Weiss et al., 2023)). The analysis presented in this work reveals that this was the case only for discontinuers, suggesting that a discontinuation confound may have contributed to a dampening of true differences between treatment condition – that is, a superior efficacy of psilocybin. That being said, it should be emphasized that discontinuers and those unmedicated on entry were equally distributed in the two study arms, and that the further refinement introduced in the analysis presented here does not invalidate the results of Carhart-Harris et al. (2021), or the conventions imposed to ensure transparency in clinical trials (i.e. pre-registration of primary outcomes). Additionally, it must be stressed that the analyses presented here were exploratory and performed post hoc in response to published comments regarding Carhart-Harris et al. (2021). Despite these limitations, they do provide interesting insights into the potential impact of discontinuation that should be considered in psychedelic clinical trials moving forward.

There are a few possible pharmacological explanations for the observed discontinuation effect. Firstly, chronic treatment with SSRI/SNRIs and related medications induce desensitization and downregulation of several serotonin receptors to maintain homeostasis (Gray and Roth, 2001). Downregulation appears to be a relatively stable phenomenon due to the time it takes for new receptors to be synthesized (Gray and Roth, 2001), rendering downregulation a possible mechanism for the current results. Particularly relevant here would be a downregulation of the 5-HT2A receptor, which is the primary receptor involved in the subjective effects of psychedelics (Becker et al., 2022; Kometer et al., 2013; Madsen et al., 2019). Previous research has identified that chronic administration of SSRI/SNRIs downregulates 5-HT2ARs in both preclinical (Klimek et al., 1994; Kubota et al., 1989; Wamsley et al., 1987) and clinical studies (Meyer et al., 2001). However, this has not been found consistently (Moresco et al., 2000; Zanardi et al., 2001). Several lines of research have highlighted the importance of the acute psychedelic experience for therapeutic effects (Roseman et al., 2017; Yaden and Griffiths, 2021); the intensity of which has been shown to correlate with 5HT2AR occupancy (Madsen et al., 2019). Thus, one natural conjecture is that a downregulation of 5-HT2ARs would cause a dampening of the acute effects of psilocybin. However, here, we found no effect of discontinuation on measures of the acute experience (mystical experiences, ego dissolutions, emotional breakthroughs and challenging experiences). This is intriguing as it could imply a dissociation between the acute pharmacological action of psilocybin (and associated subjective effects) – that is not affected by SSRI/SNRI discontinuation and an antidepressant response that is affected. Indeed, preclinical work has recently hypothesized that such a dissociation between ‘psychedelic’ subjective effects and antidepressant psychological effects is possible (Cameron et al., 2021). Additionally, recent research showed that psychological changes after psilocybin were not paralleled by a consistent change in neocortical 5-HT2AR binding induced by the treatment (one dose, assessed 1 week later), indicating that the relationship between 5-HT2AR downregulation and psilocybin’s effects may be more complex than just receptor treatment-induced changes in receptor density (Madsen et al., 2020). Moreover, given the importance of extra-pharmacological factors in shaping the acute experience (Hartogsohn, 2016), the influence of the therapeutic setting in which all dosing sessions occurred for both treatment arms should not be underestimated. Such extra-pharmacological modulation is independent of 5HT2AR downregulation. It is also important that absence of evidence is not confused with evidence of absence, and that this is explored further in future studies.

Another possible explanation of these results is that the short-term discontinuation required to enter in the trial disturbed the stability previously acquired with medications, generating discontinuation symptoms (Horowitz and Taylor, 2019). In support of this hypothesis, discontinuers showed an increase in depressive symptoms between trial screening and baseline (i.e. when withdrawal was taking place), as well as a trend towards higher depression scores at baseline compared with unmedicated patients. Tapering or cessation of SSRI/SNRIs is commonly associated with withdrawal symptoms which often resemble symptoms of depression or a worsening of depressive symptom severity (American Psychiatric Association, 2013). These symptoms can be distinguished from a relapse of the original disorder by rapidity of onset (Bhat and Kennedy, 2017; Shelton, 2001; Warner et al., 2006), the presence of somatic symptoms like nausea, shock-like feelings, dizziness (Black et al., 2000; Warner et al., 2006) and rapid response by reintroduction of SSRI/SNRIs (Bhat and Kennedy, 2017; Fava et al., 2015; Warner et al., 2006). While a minimum of 2 weeks of tapering is commonly considered sufficient (Cleare et al., 2015; National Institute for Health and Care Excellence, 2022), it may not mitigate prolonged symptoms (Horowitz and Taylor, 2019; Lerner and Klein, 2019). In fact, there is evidence suggesting that linear tapering over this period might not have additional benefit compared with abrupt discontinuation (Montgomery et al., 2004).

Withdrawal symptoms were not formally assessed over the course of the trial. However, some symptoms coded as depressive could have been elevated because in part they were elevated by antidepressant discontinuation (Hengartner and Plöderl, 2021). This may have inflated antidepressant symptomology at baseline or inflated the antidepressant action in this subgroup of patients. Reintroduction of an SSRI/SNRI after discontinuation in the escitalopram group would be predicted to mitigate withdrawal symptoms, so contributing to the greater response to escitalopram in those who discontinued.

There is growing interest in the role of expectation in shaping results of clinical trials, and psychedelic trials are no exception (Muthukumaraswamy et al., 2021). Even though they had not responded adequately, it is conceivable that those taking SSRI/SNRIs are more likely to hold SSRI/SNRIs in positive regard than those had presumably chosen to be unmedicated (despite being depressed). It was therefore important to assess whether individuals who were on SSRI/SNRIs at the time of the enrolment (and had to discontinue before starting trial treatment) had a different, more positive attitude towards conventional antidepressant medication, and whether this contributed to the discontinuation effect for the escitalopram arm. While we found no significant relationship between discontinuation and expectations of the efficacy of psilocybin, we found a trend for patients who discontinued to have higher expectation for escitalopram. This may be amplified by the conflation of withdrawal symptoms with depression symptoms discussed above, which may have been present for discontinuers when this expectation rating was collected. In the present paper, we have only addressed and discussed the relationship between expectation and discontinuation; however, a more focused and detailed analysis and discussion of expectancy effects will be presented in an upcoming paper.

Psilocybin is now being investigated for the treatment of depression, post-traumatic stress disorder, addiction, anorexia nervosa and obsessive-compulsive disorder, conditions that are often managed with serotonergic medications (American Psychiatric Association, 2010; Davis et al., 2023; Malcolm and Lee, 2017; Mithoefer, 2019; Thomas et al., 2017; Veterans Affairs Department of Defense, 2017). From a safety point of view, there is likely little risk of combining SSRI/SNRIs with classic psychedelics like psilocybin, LSD and DMT (although this is different for psychedelics containing monoamine oxidase inhibitors, like ayahuasca, which can increase the risk of serotonin syndrome (Malcolm and Thomas, 2021)). Nevertheless, discontinuation from serotonergic ADs has become convention in the field due to both a lack of fully established evidence for safety when combining psychedelics with SSRI/SNRIs, and previous reports that SSRI/SNRIs may reduce the acute effects of psychedelics (Bonson et al., 1996; Strassman, 1992), possibly reducing their therapeutic efficacy. A recent survey study also supported these earlier reports, suggesting that concurrent use of SSRI/SNRIs may weaken psilocybin’s effects acutely (Gukasyan et al., 2023), despite not investigating the sub-acute antidepressants effects. A recent small open-label trial of psilocybin therapy in 19 MDD patients taking concomitant SSRI therapy delved into the matter, finding that concomitant SSRI treatment might still lead to significant antidepressant effects after psilocybin, suggesting that discontinuation might not be required (Goodwin et al., 2023). Another RCT indicated that an atypically brief period of just 15 days of pre-treatment with escitalopram had no relevant effect on positive mood effects of psilocybin. However, it significantly reduced self-rated ‘bad’ drug effects, physiological effects and other subjective effects like feelings of ‘ineffability’, ‘any drug effect’, ‘being talkative’ and ‘open’ (Becker et al., 2022). However, both these studies had small sample sizes, and the sample of the second study consisted of healthy subjects who were pre-treated with escitalopram for just 2 weeks – likely not long enough for long-term receptor downregulation effects or other homeostatic changes. However, it is known that the team behind this study are currently undertaking a trial with 6 weeks treatment with serotoninergic antidepressants (personal communication with authors), and an increasing number of ongoing psychedelic trials are not requiring discontinuation of SSRI/SNRIs prior to study enrolment anymore.

In case future research on concomitant treatment with psilocybin and SSRI/SNRIs reveals an impairment of psilocybin’s antidepressant effects, three speculative strategies might be adopted. The first involves a cautious tapering process, potentially over longer periods as suggested by (Groot and van Os, 2018, 2021; Horowitz and Taylor, 2019), or employing partial tapering regimens aimed at reducing rather than completely discontinuing SSRI/SNRI doses. This could help minimize withdrawal symptoms and the influence of SSRI/SNRIs on the effects of psilocybin. Horowitz and Taylor (2019) note that the most substantial withdrawal symptoms occur during the final phase of tapering – from low doses to full discontinuation. However, this approach may prolong the treatment gap, increasing the risk of disease severity before psychedelic treatment. The second strategy would be to administer higher doses of psychedelics to patients currently on SSRI/SNRI treatment. This approach has not been formally researched, but a case report indicated that increasing the psilocybin dose could enable experiences akin to those with standard doses in non-SSRI/SNRI users (Strassman, 1992). Given that chronic antidepressant use may lead to 5-HT2A receptor downregulation and desensitization (Van Oekelen et al., 2003) and considering that psychedelic experience intensity correlates with 5-HT2A receptor occupancy (Madsen et al., 2019), investigating increased psychedelic doses in SSRI/SNRI-treated patients could be a promising research avenue. However, it is crucial to consider the potential for a ceiling effect in 5-HT2A receptor occupancy. It is important to note that these strategies are not mutually exclusive, and each presents a viable avenue for further investigation. Future research could explore these strategies to ascertain the most effective approach for enhancing psilocybin therapy outcomes.

Lastly, some caution in combining psilocybin with classic SSRI/SNRIs should be mentioned in cases of QTc prolongation, which can be sometimes associated with cardiac arrhythmia (Khatib et al., 2021). High doses of psilocybin could indeed lead to transient QTc increases up to 10 ms (Dahmane et al., 2021), and most common SSRI/SNRIs used at clinical doses are also associated with QTc prolongation (Beach et al., 2014). However, more research in controlled settings with chronic antidepressant users is required to better understand the interaction between SSRI/SNRIs and psychedelics before conclusions can be drawn.

### Limitations

There are several limitations that should be considered in interpreting these results. First is the small and unequal sample sizes and the exploratory, post hoc, opportunistic nature of the analyses. As such, these results should not be taken as confirmatory – rather, they may be used to inform hypothesis testing in well controlled future studies. Aside from there being a 2-week washout period at the end of tapering, the length of each medication taper was not collected during the trial and formal assessments of withdrawal symptoms were not performed, so it was not possible to include this information in our analyses. The samples that discontinued or were unmedicated at the start of the trial might have differed in other fundamental ways. For example, discontinuers may represent a more treatment-resistant form of MDD, given that they were already treated with an antidepressant medication and sought to be enrolled in a clinical trial focused on a different treatment. There is also the possibility that the unmedicated were unmedicated because they had negative expectations for SSRI/SNRIs – causing them to be primed for a negative response to escitalopram if they were allocated to that arm. Accordingly, demographic information suggests that the number of psychiatric medications previously used was higher in discontinuers than in the unmedicated. The discontinuation group also discontinued other psychiatric medications at the start of the study (see (Carhart-Harris et al., 2021) for a complete list of these medications), and this could have impacted outcomes. Finally, it is important to note that both treatment groups benefited from extensive psychological support, with an approach inspired by the Acceptance and Commitment Therapy model (Watts, 2021). The therapeutic effects derived from the support cannot thus be separated from the effects of the drugs in any of the trial arms, and likely acted synergistically to enhance both psilocybin’s and escitalopram’s effects.

## Conclusion

The current results suggest that patients with recent discontinuation of SSRI/SNRIs may be less responsive to treatment with psilocybin. If tapering is decided prior to psychedelic intervention, a more cautious approach could involve longer tapering periods (e.g. hyperbolic (Groot and van Os, 2018, 2021; Horowitz and Taylor, 2019)) and/or regimens with partial tapering focused on lowering doses instead of total discontinuation might be required. More confirmatory research as well as careful clinical consideration before deciding whether to discontinue SSRI/SNRIs is thus needed to inform future medical use. Lastly, we are mindful that a need for SSRI/SNRI discontinuation could impact on the potential future roll-out of psilocybin-therapy given that SSRI/SNRIs are the most common class of drugs prescribed for MDD, and patients tend to stay on these medications for long periods of time (Luo et al., 2020). A requirement for SSRI/SNRI discontinuation would be an inconvenience for this roll-out. Much more work is required to better understand if this is a necessary inconvenience or not.

## Supplemental Material

sj-docx-1-jop-10.1177_02698811241237870 – Supplemental material for Effects of discontinuation of serotonergic antidepressants prior to psilocybin therapy versus escitalopram for major depression Supplemental material, sj-docx-1-jop-10.1177_02698811241237870 for Effects of discontinuation of serotonergic antidepressants prior to psilocybin therapy versus escitalopram for major depression by David Erritzoe, Tommaso Barba, Meg J Spriggs, Fernando E Rosas, David J Nutt and Robin Carhart-Harris in Journal of Psychopharmacology

## Notes

1. Carhart-Harris et al. (2021) report significant differences between-treatment conditions for all secondary measures.
