# Supplementary material for: Effects of discontinuation of serotonergic antidepressants prior to psilocybin therapy versus escitalopram for major depression
Source: J Psychopharmacol. 2024 Mar 22;38(5):458–70. doi: 10.1177/02698811241237870 (PMC11102650; doi:10.1177/02698811241237870)
Supplement: sj-docx-1-jop-10.1177_02698811241237870 – Supplemental material for Effects of discontinuation of serotonergic antidepressants prior to psilocybin therapy versus escitalopram for major depression [file sj-docx-1-jop-10.1177_02698811241237870.docx]

**Supplementary information**

*Table S1*. Results of the mixed linear models with depression score (QIDS-SR16 & BDI) as outcome and time (screening, baseline) and discontinuation (unmedicated, discontinued) as predictors.

| Parameter | Estimate(SE) | t | 95% CI | p |
| --- | --- | --- | --- | --- |
| QIDS-SR16 |  |  |  |  |
| Time^ | -1.43(0.61) | -2.34 | -2.64, 0.24 | .023* |
| Discontinuation^#^ | -0.21(1.04) | -0.21 | -2.24, 1.82 | .838 |
| Time:Discontinuation | 2.21(1.03) | 2.16 | 0.21, 4.23 | .035* |
| BDI |  |  |  |  |
| Time^ | -1.04(0.86) | -1.21 | -2.72, 0.64 | 0.232 |
| Discontinuation^#^ | -0.25(1.90) | -0.13 | -3.95, 3.47 | 0.897 |
| Time:Discontinuation | 3.66(1.44) | 2.54 | 0.85, 6.48 | 0.014* |

^^^ Presented for reference time-point (*screening*)

^#^ Presented for reference condition (*unmedicated*)

* p<.05

**Table S2.** M_2_ for unmedicated patients for all outcome measures (QIDS-SR16, HAMD, MADRS, BDI, WEMWBS)**.** Note that in all cases, the factor Treatment significantly added to the model. As such, these models are examined in the main text. Effect sizes are showed under d, which reflects the ratio between the size of the estimate and the pooled standard deviation of the model . As an example, if the effect size is equal to 2, it roughly means that the estimate is two times the standard deviation of the noise.

| Parameter | | | Estimate (SD) | t | | 95% CI | | p | | d | Added effect in psilocybin (%)^#^ |  |
| --- | --- | --- | --- | --- | --- | --- | --- | --- | --- | --- | --- | --- |
| **QIDS-SR16** | | |  |  | |  | |  | |  |  |  |
| Treatment^^^ | | | -0.89(1.57) | -0.57 | | -3.93, 2.14 | | .571 | |  |  |  |
| Timepoint^^^ | | |  |  | |  | |  | |  |  |  |
|  | Week 1**^†^** | | -3.06(1.01) | -3.01 | | -5.0, -1.12 | | .003* | |  |  |  |
|  | Week 2 | | -4.23(1.01) | -4.19 | | -6.18, -2.30 | | <.001** | |  |  |  |
|  | Week 3 | | -3.89(0.96) | -4.05 | | -5.73, -2.05 | | <.001** | |  |  |  |
|  | Week 4**^‡^** | | -4.84(1.01) | -4.78 | | -6.78, -2.91 | | <.001** | |  |  |  |
|  | Week 5 | | -5.07(1.00) | -5.10 | | -6.97, -3.17 | | <.001** | |  |  |  |
|  | Week 6 | | -5.72(0.98) | -5.85 | | -7.59, -3.85 | | <.001** | |  |  |  |
|  | Follow-up | | -5.68(0.96) | -5.91 | | -7.52, -3.84 | | <.001** | |  |  |  |
| Time:Treatment | | |  |  | |  | |  | |  |  |  |
|  | Week 1:Treatment**^†^** | | -3.73(1.40) | -2.67 | | -6.40, -1.06 | | .008* | | -0.77 | 121.9 |  |
|  | Week 2:Treatment | | -2.56(1.41) | -1.82 | | -5.25, 0.13 | | .070 | | -0.53 | 60.4 |  |
|  | Week 3:Treatment | | -3.21(1.36) | -2.36 | | -5.81, -0.61 | | .020* | | -0.66 | 82.4 |  |
|  | Week 4:Treatment**^‡^** | | -3.89(1.40) | -2.78 | | -6.56, -1.22 | | .006* | | -0.80 | 80.3 |  |
|  | Week 5:Treatment | | -4.08(1.38) | -2.95 | | -6.73, -1.44 | | .003* | | -0.84 | 80.5 |  |
|  | Week 6:Treatment | | -3.39(1.37) | -2.47 | | -6.01, -0.76 | | .014* | | -0.70 | 59.1 |  |
|  | Follow-up:Treatment | | -3.00(1.36) | -2.20 | | -5.60, -0.40 | | .028* | | -0.62 | 52.8 |  |
|  | | **HAM-D** | | | | | | | | | | |
| Treatment^^^ | | | 2.10(1.18) | 1.78 | -0.18, 4.389 | |  | | .079 | |  | |
| Timepoint^^^ | | | -2.95(1.04) | -2.83 | -4.99, -0.91 | |  | | .008* | |  | |
| Time:Treatment | | | -9.52(1.49) | -6.41 | -12.43,-6.62 | |  | | <.001** -2.62 | | 323 | |
|  | | **MADRS** | | | | | | | | | | |
| Treatment^^^ | | | 1.42(2.06) | 0.69 | -2.57, 5.41 | |  | | .494 | |  | |
| Timepoint^^^ | | | -5.26(1.71) | -3.07 | -8.62,-1.91 | |  | | .004* | |  | |
| Time:Treatment | | | -12.08(2.45) | -4.94 | -16.87, -7.30 | |  | | <.001** -1.90 | | 229.5 | |
|  | | **BDI** | | | | | | | | | | |
| Treatment^^^ | | | 0.61(3.17) | 0.19 | -5.49,6.70 | |  | | .848 | |  | |
| Timepoint^^^ | | |  |  |  | |  | |  | |  | |
|  | Week 2 | | -5.28(2.27) | -2.32 | -9.64,-0.91 | |  | | .022* | |  | |
|  | Week 4 | | -6.50(2.27) | -2.86 | -10.87,-2.13 | |  | | .005* | |  | |
|  | Follow-up | | -8.55(2.27) | -3.76 | -12.92,-4.19 | |  | | <.001** | |  | |
| Time:Treatment | | |  |  |  | |  | |  | |  | |
|  | Week 2:Treatment | | -9.25(3.26) | -2.83 | -15.52,-2.99 | |  | | .006* -1.0 | | 175.2 | |
|  | Week 4:Treatment | | -13.68(3.26) | -4.19 | -19.94,-7.41 | |  | | <.001** -1.46 | | 210.4 | |
|  | Follow-up:Treatment | | -9.68(3.26) | -2.96 | -15.94,-3.41 | |  | | .004* -1.03 | | 113.1 | |
|  | | **WEMWBS** | | | | | | | | | | |
| Treatment^^^ | | | -3.58(3.00) | -1.21 | -9.50,2.13 | |  | | .229 | |  | |
| Timepoint^^^ | | |  |  |  | |  | |  | |  | |
|  | Week 2 | | -0.56(2.27) | -0.25 | -4.90,3.85 | |  | | .801 | |  | |
|  | Week 4 | | 2.38(2.35) | 1.05 | -1.82,7.22 | |  | | .297 | |  | |
|  | Follow-up | | 4.30(2.27) | 2.00 | -0.05,8.69 | |  | | .053 | |  | |
| Time:Treatment | | |  |  |  | |  | |  | |  | |
|  | Week 2:Treatment | | 13.62(3.18) | 4.29 | 7.40,19.76 | |  | | <.001* 1.48 | |  | |
|  | Week 4:Treatment | | 14.57(3.20) | 4.55 | 7.95,20.54 | |  | | <.001* 1.58 | | 527.2 | |
|  | Follow-up:Treatment | | 13.44(3.15) | 4.27 | 7.24,19.60 | |  | | <.001* 1.46 | | 311.0 | |

^ Reference condition = *Escitalopram*

# presented as percent of additional drop in QIDS-SR16 in psilocybin condition as compared to escitalopram

**^†^** Following dosing day 1

**^‡^** Following dosing day 2

* p < .05

** p < .001

**Table S3.** M_2_ for discontinuers for all outcome measures (QIDS-SR16, HAMD, MADRS, BDI, WEMWBS)**.** Note that in all cases, the factor Treatment did not add significantly to the model.

| Parameter | Estimate (SD) | *t* | 95% CI | d | p |
| --- | --- | --- | --- | --- | --- |
| QIDS-SR16 |  |  |  |  |  |
| Treatment^ | -3.89(2.55) | -1.53 | -8.75, 0.97 |  | .135 |
| Time^ |  |  |  |  |  |
| Week 1 | -6.70(1.57) | -4.26 | -9.64, -3.75 |  | < .001** |
| Week 2 | -8.44(1.57) | -5.37 | -11.38, -5.49 |  | < .001** |
| Week 3 | -9.80(1.52) | -6.43 | -12.66, -6.94 |  | < .001** |
| Week 4 | -9.03(1.57) | -5.74 | -11.98, -6.08 |  | < .001** |
| Week 5 | -8.77(1.57) | -5.58 | -11.72, -5.82 |  | < .001** |
| Week 6 | -8.20(1.52) | -5.38 | -11.06, -5.34 |  | < .001** |
| Follow-up | -7.70(1.52) | -5.05 | -10.56, -4.84 |  | < .001** |
| Time:Treatment |  |  |  |  |  |
| Week 1:Treatment | 0.70(2.14) | 0.33 | -3.32, 4.71 | 0.12 | .746 |
| Week 2:Treatment | 3.44(2.14) | 1.61 | -0.58, 7.45 | 0.59 | .111 |
| Week 3:Treatment | 3.25(2.11) | 1.55 | -0.69, 7.20 | 0.56 | .125 |
| Week 4:Treatment | 2.85(2.14) | 1.33 | -1.16, 6.86 | 0.49 | .186 |
| Week 5:Treatment | 4.23(2.14) | 1.97 | 0.21, 8.24 | 0.73 | .051 |
| Week 6:Treatment | 2.93(2.11) | 1.39 | -1.02, 6.87 | 0.50 | .167 |
| Follow-up:Treatment | 1.79(2.11) | 0.85 | -2.16, 5.74 | 0.31 | .397 |
| HAMD |  |  |  |  |  |
| Treatment^ | -1.73(2.11) | -0.82 | -5.75, 2.30 |  | .418 |
| Time^ | -8.50(2.16) | -3.94 | -12.62, -4.38 |  | < .001** |
| Time:Treatment | 0.53(3.02) | 0.18 | -5.23, 6.28 | 0.11 | .862 |
| MADRS |  |  |  |  |  |
| Treatment | 0.30(3.64) | 0.08 | -6.64, 7.24 |  | .935 |
| Time | -10.40(3.52) | -2.95 | -17.28, -3.52 |  | .008* |
| Time:Treatment | -0.12(4.93) | -0.02 | -9.82, 9.44 | -0.01 | .982 |
| BDI |  |  |  |  |  |
| Treatment^ | -2.10(5.04) | -0.42 | -11.65, 7.45 |  | .680 |
| Time^ |  |  |  |  |  |
| Week 2 | -17.88(3.41) | -5.24 | -24.28, -11.47 |  | < .001** |
| Week 4 | -16.38(3.41) | -4.80 | -22.78, -9.97 |  | < .001** |
| Follow-up | -12.50(3.41) | -3.66 | -18.90, -6.10 |  | .001* |
| Time:Treatment |  |  |  |  |  |
| Week 2:Treatment | 1.65(4.69) | 0.35 | -7.15, 10.45 | 0.16 | .726 |
| Week 4:Treatment | 4.82(4.69) | 1.03 | -3.98, 13.62 | 0.46 | .310 |
| Follow-up:Treatment | -2.39(4.69) | -0.51 | -11.19, 6.41 | -0.23 | .613 |
| WEMWBS |  |  |  |  |  |
| Treatment^ | 2.73(4.05) | 0.57 | -4.98, 10.44 |  | .505 |
| Time^ |  |  |  |  |  |
| Week 2 | 14.47(2.94) | 4.73 | 8.93, 20.01 |  | < .001** |
| Week 4 | 12.11(2.83) | 3.92 | 6.76, 17.45 |  | < .001** |
| Follow-up | 12.33(2.83) | 4.25 | 7.00, 17.67 |  | < .001** |
| Time:Treatment |  |  |  |  |  |
| Week 2:Treatment | -2.47(3.95) | -0.63 | -9.93, 4.97 | -0.27 | .534 |
| Week 4:Treatment | -3.21(3.87) | -0.83 | -10.51, 4.09 | -0.36 | .410 |
| Follow-up:Treatment | 0.12(3.82) | 0.32 | -7.07, 7.32 | -0.01 | .975 |

^ Presented for the reference condition (*Escitalopram*)

* p < .05

** p < .001

**Table S4.** Results of linear mixed model with QIDS-SR16 as outcome variable, and the following predictor variables: Treatment (escitalopram, psilocybin), Discontinuation (discontinued, unmedicated) and time (weekly from baseline to follow-up).

| Parameter | Estimate | t | 95% CI | p |
| --- | --- | --- | --- | --- |
| Treatment^ | -0.89(1.69) | -0.53 | -4.12, 2.33 | .597 |
| Discontinuation^#^ | 3.59(2.03) | 1.76 | -0.29, 7.47 | .080 |
| Time^^#^ |  |  |  |  |
| Week 1^†^ | -3.06(1.07) | -2.86 | -5.08, -1.03 | .004* |
| Week 2 | -4.24(1.07) | -3.97 | -6.26, -2.22 | <.001** |
| Week 3 | -3.89(1.01) | -3.84 | -5.82, -1.97 | <.001** |
| Week 4^‡^ | -4.84(1.07) | -4.54 | -6.87, -2.82 | <.001** |
| Week 5 | -5.07(1.05) | -4.84 | -7.06, -3.09 | <.001** |
| Week 6 | -5.72(1.03 | -5.55 | -7.67, -3.77 | <.001** |
| Follow-up | -5.68(1.01) | -5.60 | -7.61, -3.76 | <.001** |
| Time:Treatment^#^ |  |  |  |  |
| Week 1:Treatment^†^ | -3.73(1.47) | -2.53 | -6.52, -0.94 | .012* |
| Week 2:Treatment | -2.56(1.48) | -1.72 | -5.37, 0.25 | .086 |
| Week 3:Treatment | -3.21(1.44) | -2.24 | -5.93, -0.49 | .026* |
| Week 4:Treatment^‡^ | -3.89(1.47) | -2.64 | -6.68, -1.10 | .009* |
| Week 5:Treatment | -4.09(1.46) | -2.80 | -6.85, -1.32 | .005* |
| Week 6:Treatment | -3.39(1.45) | -2.34 | -6.13, -0.65 | .020* |
| Follow-up:Treatment | -3.00(1.44) | -2.09 | -5.72, -0.28 | .037* |
| Time:Discontinuation^ |  |  |  |  |
| Week 1:Discontinuation^†^ | -3.64(1.80) | -2.03 | -7.04, -0.24 | .043* |
| Week 2:Discontinuation | -4.20(1.80) | -2.34 | -7.60, -0.79 | .020* |
| Week 3:Discontinuation | -5.91(1.73) | -3.42 | -9.18, -2.63 | .001* |
| Week 4:Discontinuation^‡^ | -4.19(1.80) | -2.33 | -7.59, -0.79 | .020* |
| Week 5:Discontinuation | -3.70(1.78) | -2.07 | -7.07, -0.32 | .039* |
| Week 6:Discontinuation | -2.48(1.74) | -1.43 | -5.77, 0.81 | .154 |
| Follow-up:Discontinuation | -2.02(1.73) | -1.17 | -5.29, 1.26 | .244 |
| Treatment:Discontinuation | -3.00(2.83) | -1.06 | -0.22, 9.08 | .293 |
| Time:Treatment:Discontinuation |  |  |  |  |
| Week 1:Treatment:Discontinuation^†^ | 4.43(2.46) | 1.80 | -0.22, 9.08 | .072 |
| Week 2:Treatment:Discontinuation | 5.99(2.46) | 2.43 | 1.33, 10.66 | .015* |
| Week 3:Treatment:Discontinuation | 6.47(2.41) | 2.69 | 1.91, 11.02 | .008* |
| Week 4:Treatment:Discontinuation^‡^ | 6.74(2.46) | 2.75 | 2.09, 11.39 | .006* |
| Week 5:Treatment:Discontinuation | 8.31(2.45) | 3.40 | 3.67, 12.94 | .001* |
| Week 6:Treatment:Discontinuation | 6.31(2.41) | 2.62 | 1.74, 10.89 | .009* |
| Follow-up:Treatment: Discontinuation | 4.79(2.41) | 1.99 | 0.23, 9.35 | .047* |

^ Presented for reference condition (*Escitalopram*)

^#^ Presented for reference condition (*Unmedicated*)

**^†^** Week of dosing day 1

**^‡^** Week of dosing day 2

* p < .05

** p < .001

Table S5. Linear models for the three acute measures (Mystical Experiences Questionnaire (MEQ), Ego Dissolution Inventory (EDI) and Challenging Experiences Questionnaire (CEQ)) for the two dosing days desperately.

| Parameter | Estimate (SD) | *t* | 95% CI | p |
| --- | --- | --- | --- | --- |
| MEQ — dosing day 1 |  |  |  |  |
| Treatment | 0.47 (0.07) | 6.93 | 0.33, 0.60 | < .001** |
| Discontinuation | 0.04 (0.07) | 0.50 | -0.11, 0.18 | .622 |
| MEQ — dosing day 2 |  |  |  |  |
| Treatment | 0.39 (0.07) | 5.43 | 0.25, 0.53 | < .001** |
| Discontinuation | -0.05 (0.08) | -0.66 | -0.20, 0.10 | .514 |
| EDI — dosing day 1 |  |  |  |  |
| Treatment | 34.51 (7.29) | 4.73 | 19.90, 49.11 | < .001** |
| Discontinuation | 4.29 (7.70) | 0.56 | -11.14, 19.71 | .580 |
| EDI — dosing day 2 |  |  |  |  |
| Treatment | 31.13 (7.81) | 3.99 | 15.47, 46.80 | < .001** |
| Discontinuation | -1.01 (8.36) | -0.12 | -17.77, 15.76 | .905 |
| EBI — dosing day 1 |  |  |  |  |
| Treatment | 39.49 (7.40) | 5.34 | 24.68, 54.31 | <.001** |
| Discontinuation | 10.22 (7.81) | 1.31 | -5.43, 25.87 | .196 |
| EBI — dosing day 2 |  |  |  |  |
| Treatment | 42.71 (8.15) | 5.24 | 26.38, 59.04 | <.001** |
| Discontinuation | -0.23 (8.64) | -0.03 | -17.55, 17.09 | .979 |
| CEQ — dosing day 1 |  |  |  |  |
| Treatment | 0.15 (0.04) | 3.56 | 0.07, 0.24 | <.001** |
| Discontinuation | 0.002 (0.05) | 0.05 | -0.089, 0.093 | .959 |
| CEQ — dosing day 2 |  |  |  |  |
| Treatment | 0.25 (0.05) | 5.43 | 0.16, 0.34 | <.001** |
| Discontinuation | 0.02 (0.05) | 0.31 | -0.08, 0.11 | .756 |

* p < .05

** p < .001
